# ﻿*Carulaspissilvestrii* Lupo (Hemiptera, Coccomorpha, Diaspididae): redescription and neotype designation, with a key to *Carulaspis* MacGillivray species

**DOI:** 10.3897/zookeys.1228.138309

**Published:** 2025-02-20

**Authors:** Salvatore Nucifora, Gaetana Mazzeo, Mehmet B. Kaydan, Michele Ricupero, Agatino Russo

**Affiliations:** 1 Department of Agriculture, Food and Environment, University of Catania, Catania, Italy University of Catania Catania Italy; 2 Biotechnology Development and Research Centre, Çukurova University, Adana, Turkiye Çukurova University Adana Turkiye

**Keywords:** 28S ribosomal gene, armored scale insects, *Cupressocyparis*, identification key, *Juniperus*, molecular characterization, Sicily

## Abstract

The insect genus *Carulaspis* MacGillivray, associated with Pinidae and Magnoliidae, includes six species: *C.juniperi*, *C.minima*, *C.visci*, *C.atlantica*, *C.taxicola* and *C.silvestrii*. The first description of *C.silvestrii* lacks key morphological features that are of paramount importance for species identification. *Carulaspissilvestrii* type specimens also remain undiscovered, suggesting a potential loss of taxon information. This study provides a detailed redescription of *C.silvestrii*, including drawings, SEM images and neotype designation for taxonomic stability. For the first time, molecular characterization of *C.silvestrii* was made using a partial 28S ribosomal gene. In addition, a morphological key for distinguishing species of *Carulaspis* is provided to aid the scientific community in taxonomic and identification endeavors.

## ﻿Introduction

The genus *Carulaspis* MacGillivray, 1921 (Hemiptera: Coccomorpha: Diaspididae), is closely associated with Pinophyta (Cephalotaxaceae, Cupressaceae, Pinaceae, Taxaceae) and Santalaceae (Watson 2002; García Morales et al. 2016), and considered to be of Palearctic origin (Williams and Watson 1988; Miller and Davidson 2005; Suh and Evans 2016). The genus comprises six species, half of which are currently reported outside their area of origin, probably because they have spread with the international trade of their host plants which include ornamental plants of economic interest. These species are *C.juniperi* (Bouché, 1851), *C.minima* (Signoret, 1869), and *C.visci* (Schrank, 1781) (García Morales et al. 2016). The other species are present in a more restricted area, as follows: *Carulaspisatlantica* (Lindinger, 1911), reported in Algeria (Biche et al. 2022), Canary Islands (Lindinger 1911), Morocco (Balachowsky 1954), Spain (Gómez-Menor Ortega 1960); *C.taxicola* (Vayssière, 1913), reported only in Algeria (Vayssière 1913; Biche et al. 2022); *C.silvestrii* Lupo, 1966, reported only in Italy (Lupo 1966; Nucifora and Watson 2001).

*Carulaspissilvestrii* was collected by Professor Vincenzo Lupo (1966) on needle-like leaves and cones of *Juniperusmacrocarpa* Sm. (= J.oxicedrusL.ssp.macrocarpa Boll.) in Bosco Gravina, Donnalucata (province of Ragusa, Sicily, Italy) and dried specimens were deposited in the Department of Agriculture, Food and Environment (Di3A), University of Catania (Italy). Lupo dedicated the new species *C.silvestrii* to the entomologist Filippo Silvestri, one of the founding fathers of Italian entomology (Barbagallo 2000), and published the original species description in the journal of the Italian Academy of Entomology (Lupo 1966), which today suffers from low visibility in the international scientific community.

The original description of *C.silvestrii* lacks the description of some morphological characters that are necessary to facilitate differentiation among the other *Carulaspis* species. In addition, the name-bearing type specimens of *C.silvestrii* were never found. This study provides a detailed redescription of *C.silvestrii*, supported by drawings and photographs, with the designation of a neotype for taxonomic stability. Molecular characterization of *C.silvestrii* was also made by using a partial 28S ribosomal gene. The taxonomic position of the genus *Carulaspis* is discussed, focusing mainly on widespread species, with the aim of improving the taxonomy of the genus and providing morphological keys for species identification.

## ﻿Materials and methods

### ﻿Morphological examination

Male and female individuals of *C.silvestrii* were examined under a stereomicroscope, and details of some adult females, naturally dried and not metallised, were observed by SEM (Phenom XL G2 Desktop Thermo Fisher Scientific). Some adult females were individually mounted in Canada balsam on slides according to the method described by Watson (2002) and observed under a compound optical microscope (ZEISS Axiolab 5) at magnifications between 50 and 630×. Slide-mounted specimens of *C.silvestrii* were compared with slide-mounted specimens of *C.juniperi*, *C.minima*, *C.visci*, *C.atlantica* and with two syntypes of *C.taxicola* (Table 1).

**Table 1.** Specimens of other *Carulaspis* species studied for comparison purposes.

Species	Specimens	Host plant	Locality / Collection data	Depository
***C.atlantica*** (Lindinger)	1 adult female (HOLOTYPE)	*Juniperussabina*	Sabinosa, Canary Islands - El Hierro - Spain / 6 May 1901 - G. Lindinger *leg.*	Zoologisches Museum von Hamburg - Germany
	4 adult females (topotypes)		Sabinosa, Canary Islands - El Hierro - Spain / 13 Jul. 2023 - S. Nucifora *leg.*	Di3A, University of Catania - Italy
***C.juniperi*** (Bouché)	15 adult females	*J.hemisphaerica*	Mount Etna, Sicily - Italy / 17 Jul. 2005 - S. Nucifora *leg.*	Di3A, University of Catania - Italy
	8 adult females		Mount Etna, Sicily - Italy / 21 Mar. 2023 - S. Nucifora *leg.*	
***C.minima*** (Signoret)	13 adult females	Cupressaceaee sp.	Donnalucata, Sicily - Italy / 16 Mar. 2023 S. Nucifora *leg.*	Di3A, University of Catania - Italy
***C.visci*** (Schrank)	15 adult females	*Viscumalbum*	Mount Etna, Sicily - Italy / 21 Mar. 2023 - S. Nucifora *leg.*	Di3A, University of Catania - Italy
***C.taxicola*** (Vayssière)	2 adult females (of 5 SYNTYPES)	*Taxusbaccata*	l’Atlas de Blida - Algeria / 7 Jun. 1913 - M. Maire *leg.*	Museum National d’Histoire naturelle, Paris - France

The morphological description and terminology follow the scheme proposed by Miller and Davidson (2005). The neotype and the other studied slide-mounted specimens are deposited at the following institutions: Department of Agriculture, Food and Environment, University of Catania, Italy (**Di3A UNICT**); Muséum nationale d’Histoire naturelle, Paris, France (**MNHN**); The Natural History Museum, London, U.K. (**BMNH**); Zoologisches Museum von Hamburg, Germany (**LIB-ZMH**), and Çukurova University Scale insect collection (**CU-Sic**).

### ﻿Molecular characterization

*Carulaspissilvestrii* was molecularly characterized by sequencing ≈800 bp of the 28S gene, as proposed by Normark et al. (2019). *Carulaspisjuniperi* was included in the molecular characterization because it is considered the type species for the genus *Carulaspis* and has closer morphological similarities to *C.silvestrii*. DNA was extracted from three sets of 30 specimens each using the E.Z.N.A.^®^ Tissue DNA Kit (Omega Bio-tek, Inc., Norcross, GA, USA) with a destructive extraction protocol. Primer pairs were 28s_s3660 5’- GAG AGT TMA ASA GTA CGT GAA AC -3’ and 28s_a335 5’- TCG GAR GGA ACC AGC TAC TA -3’ as described in Normark et al. (2019). PCR was carried out according to the protocol described in Ricupero et al. (2021) with slight modifications. Each reaction was performed in 20 μL volume with 0.85X FailSafe^TM^ PCR 2X Pre-Mix F (Lucigen Corporation, Middleton, WI, USA), 0.5 μM of each primer, 1.5 units of Taq DNA Polymerase (Invitrogen, Thermo Fisher Scientific, Waltham, MA, USA) and 2 μL of DNA template. The cycling conditions were: 96 °C for 5 min, 35 cycles at 96 °C for 65 s, 45 °C for 1 min, 72 °C for 1 min, followed by a final cycle at 72 °C for 10 min. Reactions and cycling conditions were performed in Applied Biosystems^TM^ MiniAmp^TM^ Plus thermal cycler. PCR amplificates were first checked by electrophoresis on 1% agarose gel. Successful PCR products were then purified and sequenced by BMR Genomics sequencing service (Padova, Italy) using the Sanger method. For each species, the coding regions were checked for errors and trimmed for low quality. Since the sequences were identical, they were merged into a single one using Unipro UGENE version 1.26.1. The resulting FASTA files were aligned to reference sequences from the National Center for Biotechnology Information (NCBI) GenBank^®^ using the Basic Local Alignment Search Tool for species identification (Altschul et al. 1990). The sequences of *C.juniperi* and *C.silvestrii* were deposited in GenBank under the following accession numbers: PP910166 and PP910167, respectively.

## ﻿Taxonomy

### ﻿ Carulaspis silvestrii

Taxon classificationAnimaliaHemipteraDiaspididae

Lupo, 1966

7F90060B-072B-5C95-B093-55452CE7A8C8

**Figure 1.**
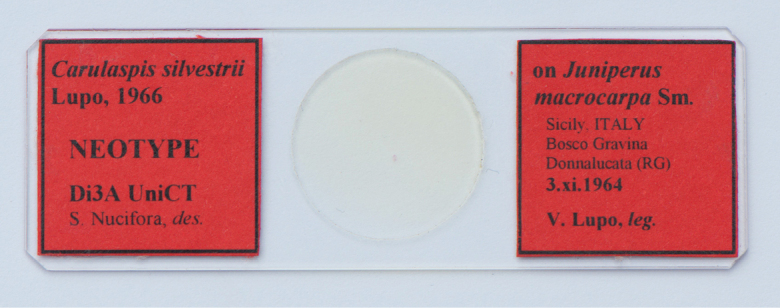
The slide of the neotype of *Carulaspissilvestrii* Lupo, 1966.

**Figure 2.**
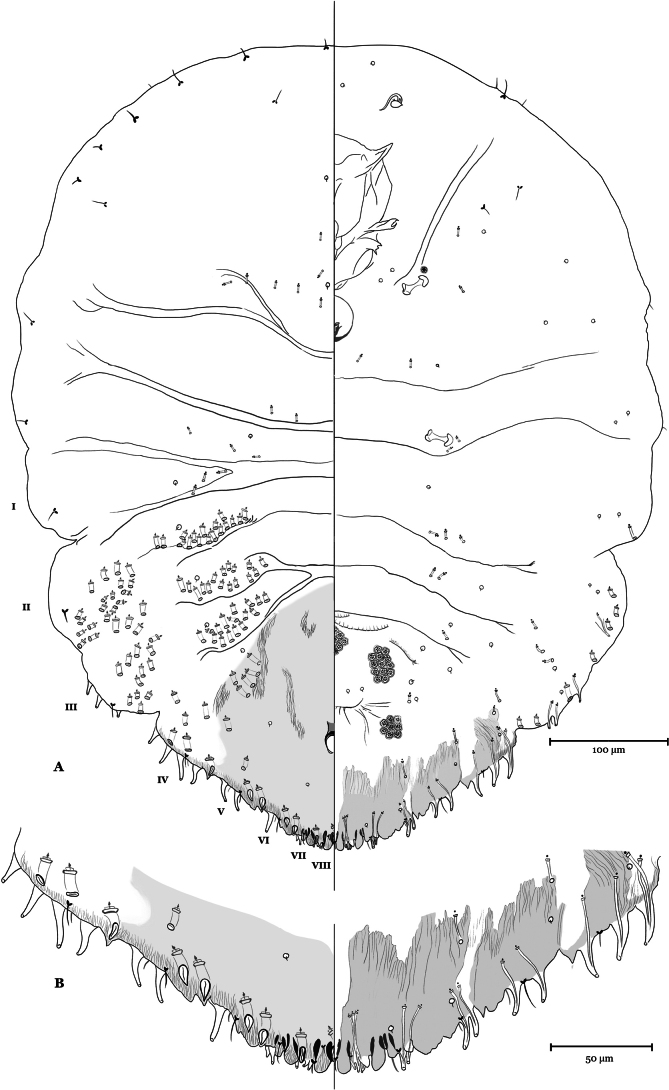
*Carulaspissilvestrii* Lupo, 1966, neotype, adult female showing features of the dorsum on the left side and those of the venter on the right **A** habitus **B** detail of pygidial margin and submargin.

**Figure 3.**
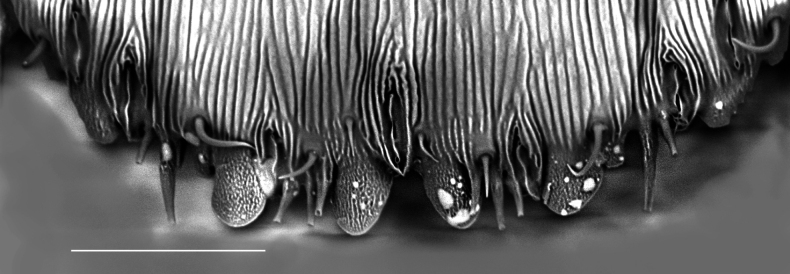
*Carulaspissilvestrii* Lupo, 1966, specimen showing detail of pygidial margin, dorsal side, segments 6–8. Scale bar: 25 µm. (a few drops of hardened glue are visible on the sample).

**Figure 4.**
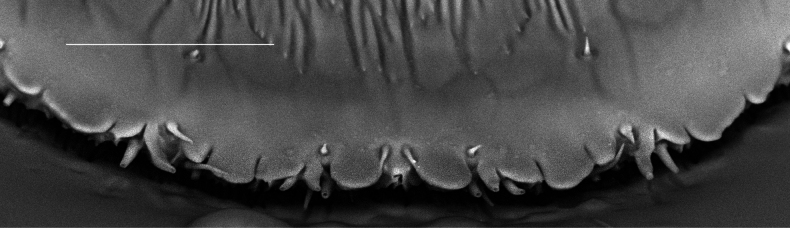
*Carulaspissilvestrii* Lupo, 1966, specimen showing detail of pygidial margin, ventral side, segments 6–8. Scale bar: 25 µm.

**Figure 5.**
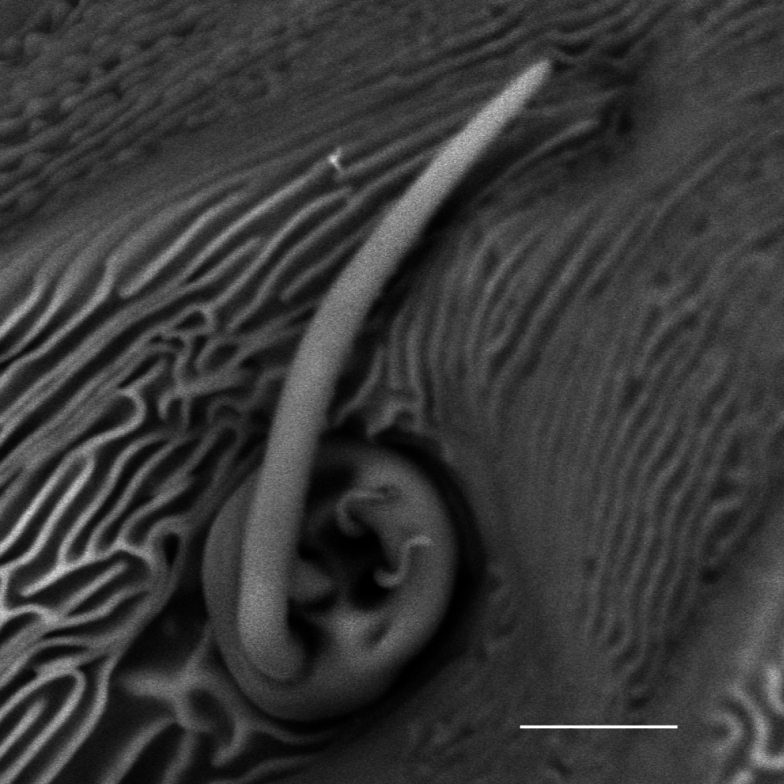
*Carulaspissilvestrii* Lupo, 1966, specimen showing antenna. Scale bar: 5 µm.

**Figure 6.**
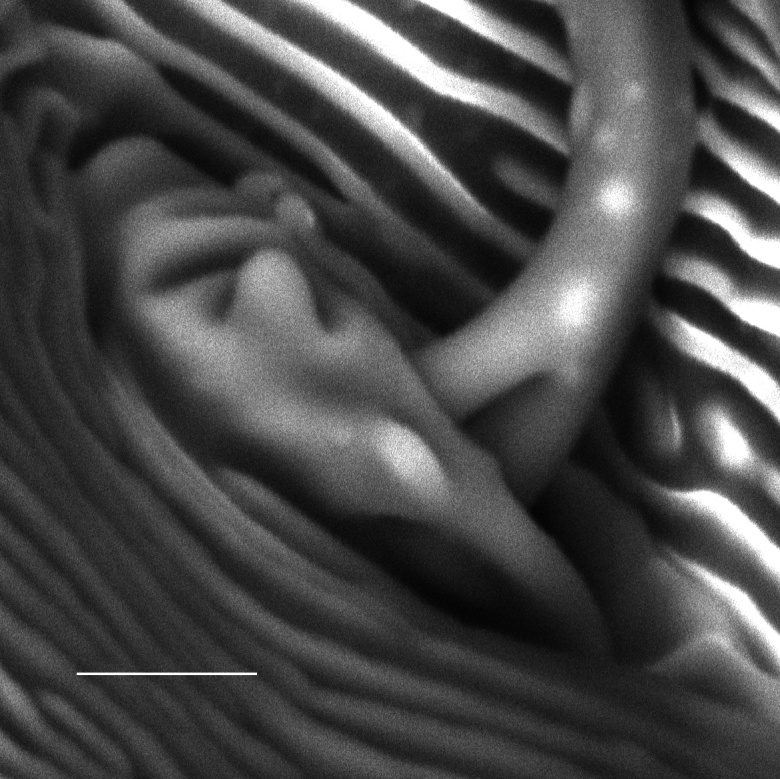
*Carulaspissilvestrii* Lupo, 1966, specimen showing antenna detail. Scale bar: 2.5 µm.

Figs 1 , 2 , 3 , 4 , 5 , 6

#### Neotype designation.

Having established that there are no type specimens of *Carulaspissilvestrii* Lupo, 1966 that bear the name, a neotype of this taxon was designated in accordance with the provisions of Article 75 of The International Code of Zoological Nomenclature (International Commission on Zoological Nomenclature 1999). The qualifying reasons for this proposal are as follows: (i) the neotype is designed to clarify the taxonomic status of *C.silvestrii* compared to other species of the same genus; (ii) data (i.e., labelled text on the permanent microscopic slide of the neotype) and morphological description of *C.silvestrii* given in the present paper are appropriate to ensure recognition of the specimen here designed as neotype; (iii) most co-authors of the present publication belong to the Department of Agriculture, Food & Environment (formerly Faculty of Agriculture), University of Catania, where Prof. V. Lupo worked and where his collection of Diaspididae scales is deposited; they acquired here the certainty that the name-bearing type specimens of *C.silvestrii* have never been found, so assuming they were lost; (iv) the proposed neotype is designated from the type locality and host plant from which this species was recorded; it is obtained from a dry original sample collected and labelled by Prof. V. Lupo on November 3, 1964, mounted by us on a permanent microscopical slide using Canada balsam medium; (v) it is thereby declared that the designated neotype is the property of the University of Catania, where it is preserved at the Section of Applied Entomology, the Department of Agriculture, Food & Environment (Di3A), where a research entomological collection is available, with facilities for the preservation of name-bearing type specimens which are accessible for their study.

#### Material examined.

***Neotype*** (here designated): Italy, Sicily • adult female, neotype of *Carulaspissilvestrii*; right label [red label]: on *Juniperus* / *macrocarpa* Sm. / Sicily, ITALY / Bosco Gravina [currently part of The Irminio River Nature Reserve] / Donnalucata (RG) / 3.xi.1964 / V. Lupo leg.; left label [red label]: *Carulaspissilvestrii* / Lupo, 1966 / NEOTYPE / Di3A UniCT / S. Nucifora, des.; deposited at Di3A UNICT (Fig. 1).

#### Other material.

Italy, Sicily • 13 adult females (topotypes); 3 Nov. 1964; Ragusa province, Donnalucata, Bosco Gravina; V. Lupo leg.; on *Juniperusmacrocarpa*; 8 specimens deposited at Di3A UNICT, 1 specimen at LIB-ZMH, 1 specimen at MNHN, 1 specimen at BMNH, 2 specimens at CU-Sic • 2 adult females (topotypes); 7 Oct.1997; Ragusa province, Donnalucata, The Irminio River Nature Reserve; S. Nucifora leg.; on *J.macrocarpa*; deposited at Di3A UNICT • 2 adult females (topotypes); 16 Jan. 2023; S. Nucifora leg., Ragusa province, Donnalucata, The Irminio River Nature Reserve; 36°46.46'N, 14°35.68'E; alt. ca 5 m a.s.l.; on *J.macrocarpa*; deposited at Di3A UNICT • 5 adult females; 16 Mar. 2023; S. Nucifora leg.; Ragusa province, Donnalucata; 36°46.58'N, 14°36.42'E; alt. ca 18 m a.s.l.; on × *Cupressocyparisleylandii* (A.B. Jacks. & Dallim.) Dallim (new host association); deposited at Di3A UNICT.

#### Redescription of *Carulaspissilvestrii* adult female.

***Appearance in life*.** Adult female cover rather circular, slightly convex, white; with central exuviae, yellow; ventral veil often absent, sometimes reduced to a thin white partial residue. Male cover elongate, white, felted and with 1 or 3 longitudinal ridges, with apical exuvia, yellow. Body of adult female yellow or reddish-brown. On needle-like leaves and cones.

***Slide-mounted characters of the adult female*** (Figs 2–6). Body oval, longer than wide, slightly turbinate. The greatest width of the body is generally at the level of metathorax. Segment 1 of the abdomen and the thorax generally of similar width. Stronger intersegmental narrowing at margin between abdominal segments 1–2 and 3–4. The first and second pairs of ***lobes*** well-developed; third and fourth lobes represented by sclerotized raised areas. Median lobes not yoked, generally a little bit longer than wide, with medial and lateral margins parallel or diverging slightly, without evident notches, separated by space 0.4–0.9 times width of median lobe. Second lobes bilobed, without notches, with medial lobule about same size as median lobe. Third lobes reduced to a projecting ridge, sometimes with few light serrations on lateral margin, with two rounded apices as trace of lobules, without medial notches. Fourth lobes as a marginal thickening with only one rounded distal apex, sometimes with a light trace of lobules and with few small serrations on lateral margin; small ***paraphyses*** on the developed lobes. Dorsal seta laterad of median lobes never extending beyond apex of lobes. Anal opening located 2.2–4.0 times length of anal opening from base of median lobes, anal opening 8–13 µm long. ***Macroducts*** located in body margin and submarginal and submedial areas of abdomen, with total of 50–120 ducts on each side of body, counting all macroduct orifices anterior to anal opening. Dorsal macroducts of 2 sizes, larger size macroducts present in marginal areas of segments 4 to 8, smaller size macroducts located near body margin in submarginal areas and in submedial areas of segments 2–5, generally absent on submarginal and submedial areas of segments 6 or 7; with one macroduct between median lobes 10–13 µm long, one macroduct in first space 11–15 μm long, with 8–13 on each side of pygidium on segments 5 to 8. ***Microducts*** in dorsal surface located in medial area among head and prothorax, with a cluster of 7–13 ducts and in medial or submedial areas, from mesothorax to segment 1, and with 5–10 ducts on each side of body. Pygidial microducts absent on dorsum. ***Ventral microducts*** on pygidium located in submarginal areas of segments 5 and 6, with 2–4 ducts on each side of body, and in submedial areas on segment 5, at level of the vulva, with 1 or 2 ducts, on each side of body; present in medial and submedial areas of thorax and prepygidium, with 9–16 ducts. ***Macroducts in ventral side***, similar to small-size macroducts on dorsum, located on margin and submargin of any of segments 3, 2 or 1. ***Vulva*** opening lies 0.5–0.6 times of the length of the ventral side of the pygidium at the beginning of the free margin. ***Perivulvar pores***, in 5 rounded groups, 19–34 pores on each side of body. ***Crenulae*** present on ventral side of pygidium in median position, anteriorly to the vulva opening. Hardly noticeable crenulae sometimes present on medial area of prepygidial sternites. ***Spiracles*.** Anterior, with 1 or 2 perispiracular pores, posterior without pores. ***Antennae*.** Tubercle-like, with 2 short, thin setae, and 1 conspicuous curved seta, which is basally divided into 2 setae (Figs 5–6). ***Gland-spine*** formula mainly 3-2-1; on first space rarely present only 2 gland spines, on second space occasionally only 1 and on third space sometimes 2. Other marginal gland spines on each side of body are: 1–3 on segment 5; 2–4 on segment 4; 1–5 on segment 3 with some element sometimes in submarginal position; submarginal gland spines 0–3 ventrally on segments 2, with some element occasionally in marginal position. Rarely, gland spine with two glandular ducts. Without gland spines between median lobes. ***Eyes*** absent.

#### Comments.

In the genus *Carulaspis*, the more conspicuous antennal seta of the adult female is divided at the base (Figs 5, 6); it remains to be verified whether this is also the case in *C.taxicola*. For the latter, only a limited number of incompletely detailed photographs of two syntypes were available for observation. In Diaspididae, such an antennal feature is reported at least for: *Crassaspismultipora* (Ferris), *Diaspistexensis* (Cockerell), *Furchadaspiszamiae* (Morgan) (Ferris 1937); *Getulaspisbupleuri* (Marchal), *Voraspisceratoniae* (Marchal) and *Voraspisnerii* (Newstead) (Balachowsky 1954). Based on some occasional observations, it was found in: *D.boisduvalii* Signoret, *D.bromeliae* (Kerner), *D.echinocacti* (Bouché), *Epidiaspisgennadii* (Leonardi), *E.leperii* (Signoret) and *Mohelnaspisampelodesmae* (Newstead).

*Carulaspissilvestrii* was confirmed on the host indicated by Lupo (1966) and recorded as a new host association on Leyland cypress, × *Cupressocyparisleylandii*.

*Carulaspissilvestrii* is close to *C.juniperi* and somewhat similar to *C.visci* and *C.minima*; it differs in having the body of the adult female more elongate and less turbinate, in the presence of three gland spines on the first spaces (between the median lobe and the second lobe), the absence of gland spines between median lobes. The gland spines between the median lobes are also lacking in *C.atlantica*, which generally looks more distant from its congeners; it has a rounder body and different size and shape of median lobes. *Carulaspissilvestrii* differs from *C.atlantica* and *C.visci* also by the absence of submarginal dorsal macropores after segment 5 in adult females, and from *C.minima* in having a median macropore between the L1. The body shape of the adult female of *C.taxicola* greatly differs from that of *C.silvestrii*, with the prosoma and prepygidium tending to be wider, and the pygidium particularly acute. In addition, the number of marginal and submarginal ducts on the first abdominal segment is different in the two species.

The morphological similarity of adult females of the genus *Carulaspis* and the vagueness of their descriptions by previous authors have led to great nomenclatural confusion and unclear definitions (Danzig 1993). In addition, the intraspecific variability of its species may overlap among taxa, such as the number and arrangement of marginal and submarginal macroducts (Boratyński, 1957) and of gland spines, which show a small range of variability in number, which is reduced and becomes exceptional or null in the last segments of the pygidium.

#### Molecular characterization.

Based on 28S sequence data, *C.silvestrii* and *C.juniperi* collected from different hosts in Sicily shared a haplotype with 99.02% identity. The resulting sequences were aligned to reference sequences from NCBI and compared with publicly available data on GenBank yielding an identity score of 99.2% and E-value = 0.0 with *C.juniperi* isolate from USA (Accession number DQ145301.2).

### ﻿Key to adult females of *Carulaspis* species

While emphasising that a rather holistic taxonomic approach is useful for the morphological identification of species in the genus *Carulaspis*, we conclude that the species currently included in the genus are valid: each has its own morphological distinguishing characters, some of which are included in the following identification key.

**Table:** 

1(0)	With more than 3 dorsal ducts on each side of the marginal and submarginal area of first abdominal segment	***Carulaspistaxicola* (Vayssière)**
–	With 0–3 ducts on each side of the marginal and submarginal area of first. abdominal segment	**2**
2(1)	With a single gland spine between second lobe (L_2_) and median lobe (L_1_). L_2_ not clearly bilobular, significantly larger than L_1_	***Carulaspisatlantica* (Lindinger)**
–	With more than one gland spine between L_2_ and L_1_. L_2_ clearly bilobular; medial lobule size close to L_1_.	**3**
3(2)	With 3 gland spines between L_2_ and L_1_; without gland spines between median lobes	***Carulaspissilvestrii* (Lupo)**
–	With 2 gland spines between L_2_ and L_1_; with 2 gland spines between median lobes	**4**
4(3)	With marginal macroduct between median lobes	**5**
–	Without marginal macroduct between median lobes	***Carulaspisminima* (Signoret)**
5(4)	With submarginal macroducts on segment 6	***Carulaspisvisci* (Schrank)**
–	Without submarginal macroducts on segment 6	***Carulaspisjuniperi* (Bouché)**

## ﻿Conclusions

The redescription of *Carulaspissilvestrii* showed morphological details of the adult female that were not sufficiently highlighted in the original description, and the definition of the species has been enriched by molecular characterization. These acquisitions will allow the species to be correctly identified. *Carulaspissilvestrii* which is currently known only from Sicily, could have a much wider distribution in Italy and the Mediterranean, depending on the distribution of its host plants on which the armored scale can cause severe infestations, with possible economic consequences.

## Supplementary Material

XML Treatment for Carulaspis silvestrii

## ﻿Additional information

### Author ORCIDs

Salvatore Nucifora https://orcid.org/0000-0002-3627-683X

Gaetana Mazzeo https://orcid.org/0000-0002-6676-8077

Mehmet B. Kaydan https://orcid.org/0000-0002-0677-255X

Michele Ricupero https://orcid.org/0000-0001-5392-7553

Agatino Russo https://orcid.org/0000-0002-6949-397X

### Data availability

All of the data that support the findings of this study are available in the main text.
